# Identification and distribution of the NBS-LRR gene family in the Cassava genome

**DOI:** 10.1186/s12864-015-1554-9

**Published:** 2015-05-07

**Authors:** Roberto Lozano, Martha T Hamblin, Simon Prochnik, Jean-Luc Jannink

**Affiliations:** Department of Plant Breeding and Genetics, Cornell University, Ithaca, NY 14853 USA; Institute for Genomic Diversity, Biotechnology Building, Cornell University, Ithaca, NY 14853 USA; US Department of Energy, Joint Genome Institute, Walnut Creek, CA 94598 USA; United States Department of Agriculture, Agricultural Research Service (USDA-ARS) R.W. Holley Center for Agriculture and Health, Ithaca, NY 14853 USA

## Abstract

**Background:**

Plant resistance genes (R genes) exist in large families and usually contain both a nucleotide-binding site domain and a leucine-rich repeat domain, denoted NBS-LRR. The genome sequence of cassava (*Manihot esculenta*) is a valuable resource for analysing the genomic organization of resistance genes in this crop.

**Results:**

With searches for Pfam domains and manual curation of the cassava gene annotations, we identified 228 NBS-LRR type genes and 99 partial NBS genes. These represent almost 1% of the total predicted genes and show high sequence similarity to proteins from other plant species. Furthermore, 34 contained an N-terminal toll/interleukin (TIR)-like domain, and 128 contained an N-terminal coiled-coil (CC) domain. 63% of the 327 R genes occurred in 39 clusters on the chromosomes. These clusters are mostly homogeneous, containing NBS-LRRs derived from a recent common ancestor.

**Conclusions:**

This study provides insight into the evolution of NBS-LRR genes in the cassava genome; the phylogenetic and mapping information may aid efforts to further characterize the function of these predicted R genes.

**Electronic supplementary material:**

The online version of this article (doi:10.1186/s12864-015-1554-9) contains supplementary material, which is available to authorized users.

## Background

In the tropics, cassava (*Manihot esculenta*) is the third biggest source of carbohydrates after rice and maize, feeding almost a billion people daily (www.fao.org). Most importantly, it is one of the major food crops in sub-Saharan Africa. However, two viral diseases threaten cassava productivity: Cassava Mosaic Disease (CMD) and Cassava Brown Streak Disease (CBSD) [1,2]. While these viruses were previously associated with lowlands, a new variant of the Cassava Brown Streak virus was found recently to infect this crop at altitudes above 1000 m [3]. In Uganda, the disease is pandemic, and its devastating effects, makes this virus a major concern for food security in central and east Africa [4]. Because of these diseases, an understanding of the molecular basis of disease resistance in cassava is a priority. As a first step, we have used the cassava genome sequence to identify and classify members of a major class of disease-resistance genes.

Studies in model plant species have shown that, unlike vertebrates, plants lack a somatic adaptive immune system [5]. To resist pathogens, plants have developed an advanced innate immune system consisting of a multiple layered network of defense proteins. One of these layers, Effector-Triggered Immunity (ETI), acts inside the cell via proteins encoded by a class of defense genes called R genes [6]. The most common disease resistance genes cloned to date are those belonging to the NBS-LRR family, named after the domains they typically contain: the nucleotide binding sites (NBS) and the leucine-rich repeat (LRR). This highly conserved gene family has structural and functional homology to the mammalian nucleotide-binding oligomerization domain (NOD)-LRR protein family, which functions in inflammatory and immune responses [7,8].

The NBS domain is part of the larger ~300 amino acid NB-ARC domain and contains strictly ordered motifs [9]. The NBS region binds and hydrolyzes ATP and GTP and primarily works as a signal transduction switch following pathogen recognition. LRR domains typically consist of 20–30 amino acid repeats that are often implicated in protein-protein interaction and, more precisely, bind to pathogen-derived molecules [10]. The LRR domain is thought to be the primary determinant of pathogen recognition specificity [11-13]. NBS-LRR proteins can recognize a wide variety of taxonomically unrelated pathogens, including viruses, bacteria, fungi, and even insects [14]. Activation of these genes results in a hypersensitive response (HR), a localized form of host-programmed cell death [15].

Resistance genes encoding NBS domains can be further classified into two major groups according to the presence or absence of different domains in the N-terminal region. The first group is comprised of proteins carrying the TOLL/interleukin-1 receptor (TIR) and are named TNL proteins (for TIR-NBS-LRR). The second, non-TIR-NBS-LRR group is usually known as CNL (for CC-NBS-LRR), because most of its members encode a coiled-coil (CC) N-terminal domain. Despite the name, we can find members of this group with Zinc finger or RPW8 domains instead of a coiled-coil [16-18]. This division is reflected in both phylogenetic analysis and their signaling pathways [19]. Both TIR and CC domains are involved in downstream specificity and signaling regulation [20].

While molecular techniques can be used to analyse NBS-LRR genes in plants lacking a genome sequence [21], the increasing number of sequenced plant genomes has facilitated the study of the NBS-LRR family in dicots and monocots, including *Arabidopsis thaliana* [22], *Arabidopsis lyrata* [23], *Oriza sativa* [24,25], *Vitis vinifera* [26], *Glycine max* [27], *Malus domestica* [28], *Solanum tuberosum* [29,30], and *Solanum lycopersicum* [31]. In most of these studies, the NBS-LRR genes exist in large, diverse families that are clustered on the genome [32,33].

The genomic clustering of R genes is thought to facilitate rapid R gene evolution in plant genomes via recombination. These clusters vary in size and complexity and fall into two types based on the phylogenetic relationship of their members. Commonly, clusters contain closely-related genes (same recent ancestor) of the same type, but they can also be heterogeneous, with NBS-LRR genes that are phylogenetically distant from each other (i.e., clusters can contain both TNL and CNL genes) [14,34].

In a recent effort to accelerate functional R gene discovery in cassava, several Resistant Gene Analogs (RGA) were identified using molecular techniques [35]. The *Manihot esculenta* genome comprises 12,977 scaffolds (L50 = 258,147 bp) [36] and together with gene annotations, and the genetic map [37], represent powerful tools for identifying and mapping resistance genes.

Among the 30,666 annotated protein-coding genes, we identified 228 belonging to the NBS-LRR family. Annotation of functional domains, physical position, as well as expression profiling and phylogenetic analysis was performed on these genes. Our results provide significant insights into the evolution of this gene family in the cassava genome, and the results also generated an extensive R gene database that will accelerate future efforts for disease resistance breeding in this crop.

## Methods

### Cassava genome resources

The whole v4.1 genome assembly of the AM560-2 genotype comprising 12,977 scaffolds, as well as the whole genome annotation (30,666 genes), were downloaded from Phytozome [38] (http://www.phytozome.net/ accessed on 01/24/2014). Subsequently, a genetic map was used to anchor scaffolds from v4.1 onto the genetic map, creating 18 pseudomolecules (*M. esculenta* v5.0, http://phytozome.jgi.doe.gov).

### Identification of NBS-LRR genes

Predicted proteins from the cassava genome were scanned using HMMER v3 [39] using the Hidden Markov Model (HMM) corresponding to the Pfam [40] NBS (NB-ARC) family (PF00931; http://pfam.sanger.ac.uk/). From the proteins obtained using the raw NBS HMM, a high-quality protein set (E-value < 1 × 10^−20^ and manual verification of an intact NBS domain) was aligned and used to construct a cassava-specific NBS HMM using hmmbuild from the HMMER v3 suite. This new cassava-specific HMM was used, and all proteins with an E-value lower than 0.01 were selected. NBS-LRR genes were further filtered based on manual curation and functional annotation against both the closest homolog from *Arabidopsis* and the UNIREF100 sequence database. Most of the proteins that were removed had at least a partial kinase domain, but no relationship to NBS-LRR genes; this result was expected because the NBS domain has smaller kinase subdomains (Additional file 1).

### NBS-associated conserved domains

NBS-encoding resistance genes usually have additional domains such as TIR, CC, or RPW8 in the N-terminal domain and a variable number of LRR domains in the carboxy-terminal region [5]. Conserved, associated domains were identified using a hmmpfam comparison to Pfam v27 [40]. The raw TIR HMMs (PF01582), RPW8 (PF05659), and LRR (PF00560, PF07723, PF07725, and PF12799) were downloaded (http://pfam.xfam.org) and used to mine the previous NBS-encoding gene candidates to identify distinct domains. Results were confirmed using both the NCBI Conserved Domains Tool [41] and Multiple Expectation for Motif Elicitation (MEME) [42]. Paircoil2 was used [43] with a P score cut-off of 0.03, because coiled-coil domains cannot be identified through conventional Pfam searches (Additional file 2).

### Identification of partial NBS-LRR genes

Due to the rapid evolution of the NBS-LRR family, our pipeline might not identify some genes that belong to the NBS-LRR cluster, but which have lost the NBS domain, or a large part of it. To try to identify all of these genes, we used an in-house script to download all the proteins from NCBI that included an “NBS-LRR” tag in their names. Later these proteins were formatted as a BLAST database. The remaining proteins from the cassava annotation were searched with BLAST [44] against this database. We kept high similarity genes as partial genes that could be pseudogenes caused by deletion, insertion, or frameshift mutation.

### Alignment and phylogenetic tree estimation

We conducted this analysis to confirm the separation between the two main NBS-LRR groups in cassava and to learn about the phylogenetic history of the genes within each main branch. The NB-ARC domain region for every protein that carried a full-length NBS, as revealed by MEME [42], was extracted (counting 250 aa after the p-loop). Sequences with less than 90% of the full-length NB-ARC domain were excluded from posterior analysis. The multiple alignment was performed using clustalW [45] on 157 full NBS-domain cassava genes under default parameters. The resulting alignment was manually curated using Jalview [46], and poorly aligned regions at both ends were trimmed. A phylogenetic tree was then inferred in MEGA6 [47] by using the Maximum Likelihood method based on the Whelan and Goldman + freq. Model [48]. The tree with the highest log-likelihood was selected. Initial trees for the heuristic search were obtained by applying the Neighbour-Joining method to the matrix of pairwise distances estimated using a JTT model [49]. The nodes were tested by bootstrap analysis with 1000 replicates. Two additional trees were constructed using the same methodology, but which included reference resistance NBS-LRR genes from other species (Additional file 3). All trees were rooted using the NBS domain of the Human apoptotic protease-activating factor-1 (APAF-1).

### Anchoring NBS-LRR genes to the cassava pseudomolecules

The NBS-LRR candidate genes were mapped to their physical position in the cassava genome using the cassava pseudomolecule assembly v5 and the genes from annotation v4.1 (Phytozome, http://phytozome.jgi.doe.gov). Genes were mapped to their position in the pseudomolecule file using Blast + [50]. Only the top hit was considered (full coverage of both query and subject). CIRCOS [51] and Mapchart [52] were used for visualization.

Genes were arranged in different clusters. As described previously [29], an NBS-LRR cluster is defined as two or more NBS-LRR genes that are closer than 200 kb and separated by no more than eight non-NBS-LRR genes. To test the statistical significance of this definition, we compared the cluster frequencies of NBS-LRR genes with the mean cluster frequencies obtained from 1000 iterations of a random sample of genes. Each random sample consisted of 205 genes, which is the same number of NBS-LRR genes that is anchored to its chromosomal positions.

### Expression analysis of NBS genes under biotic stresses

RNAseq data were obtained from two experiments. The first measured changes in the transcriptome after infecting plants with Cassava Brown Streak Virus (CBSV) [53]. This study was focused on detecting genes involved in the steady state defence response by carrying out a transcriptome analysis 12 months after graft inoculation of CBSV. In the experiment, leaf samples were collected from three CBSV-inoculated and control plants, and two cassava genotypes were used, Kaleso (resistant to CBSV) and Albert (susceptible). RNA was sequenced using the Illumina Hiseq 2000 platform to generate 50 bp single end reads. BWA aligner was used to map the reads against the cassava genome. FPKM (Fragments per Kilobase of exon per Million fragments mapped) were calculated for each gene, but only transcripts showing an FPKM > 1 were kept for further analysis. Differential expression was calculated using the R package DEGseq [54].

The second RNAseq experiment measured the transcriptome response of a susceptible cassava plant (cultivar MCOL1522) infected with both a pathogenic and a non-pathogenic strain of *Xanthomonas axonopodis* pv. Manihotis (causal agent of Cassava Bacterial Blight) [55]. Two biological replicates were performed and RNA samples were collected at 0, 5, and 7 days post inoculation. RNAseq was run using Illumina technology to give 100 bp pair-end reads. FPKM values for all annotated cassava genes were obtained using cufflinks v2.0.2 [56]. Differential expression was calculated using NOISeqBIO v2.6.0 [57].

Differentially expressed genes from both experiments were scanned for NBS-LRR genes. It was expected that these genes were overexpressed during infection if they were contributing to the response against the pathogens.

### Availability of supporting data

Phylogenetic raw data are available through the Data Dryad digital repository, doi:10.5061/dryad.tp030. Nucleic acid and protein sequences for every gene presented in this article are available in the Phytozome v10.1 repository, http://phytozome.jgi.doe.gov (Manihot esculenta v4.1).

## Results

### Identification of NBS-LRR genes

The cassava-specific HMM for the NBS-LRR domain identified 490 gene candidates. This initial dataset was filtered based on several criteria (Additional file 1). Finally, a total of 228 non-redundant NBS-encoding R gene candidates were identified in the v4.1 release of *Manihot esculenta* genome, as well as 99 partial genes (without the NBS domain) (Table 1, Additional file 4). Analysing each NBS-LRR candidate allowed us to classify them into the TNL or CNL families (Table 1). Proteins belonging to the CNL group include 117 with full-length domains (CC, NBS and LRR). However, 64 proteins from this group lacked a domain and were classified as follows; N_CC_ (10, only NBS domain from the CC type), CN (11, N terminal domain and NBS, but lacking the LRR), NL_CC_ (43, NBS and LRR from the CC type, but lacking the N terminal domain). The remaining 47 genes belonged to the TNL group and were distributed as follows: TNL (29), N_TIR_ (4), TN (5), NL_TIR_ (9).

**Table 1:** **NBS-LRR genes and their classification in different genomes**

**Predicted domains**	**Letter code**	***Manihot esculenta***	***Oriza sativa***	***Populus trichocarpa***	***Arabidopsis thaliana***
**CNL Type**					
NB	N_CC_	10	25	27	1
CC NB	CN	11	7	19	5
CC NB LRR	CNL	117	160	119	51
NB LRR	NL_CC_	43	16	71	4
Other	-	N/A*	264	43	1
**TNL Type**					
NB	N_TIR_	4	-	19	-
TIR NB	TN	5	3	13	21
TIR NB LRR	TNL	29	-	64	83
NB LRR	NL_TIR_	9	-	12	2
Other	-	N/A*	N/A*	15	39
Partial	P	99	60	N/A*	N/A*
**Total**		**327**	**535**	**402**	**207**
# of genes in the genome		30666	37544	45550	25498
Genome size		~760 Mb	389 Mb	485 Mb	125 Mb

Source: This article IRGSP (2005); (Yang et al. [26]); (Meyers et al. [22]); (Zhou et al., [25]).

*Not Analyzed.

The average number of exons among the full-length NBS-encoding genes (CNL and TNL) in the cassava genome was 3.35, a value that is approximately half the average number of exons among all predicted cassava genes (6.17). As expected from previous studies, the average number of exons from the TNL family was higher (5.48) than those in CNL genes (2.82). Moreover, 35% of all the CNL genes were encoded by a single exon. This result is consistent with *Arabidopsis thaliana*, *Malus domestica,* or *Brassica rapa*, where CNLs and TNLs have 2.2, 2.3, 3.4, and 5.3, 5.2, 6.4 exons per gene on average, respectively [18,22,28].

### Phylogenetic analysis

**Figure 1:**
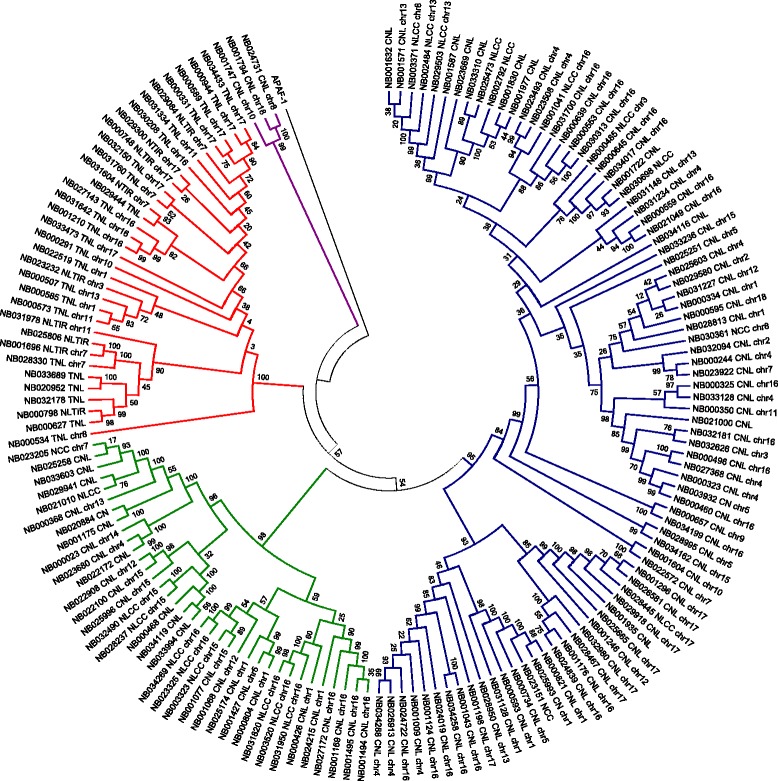
Phylogenetic reconstruction of the NBS-LRR proteins in *Manihot esculenta*. A maximum likelihood tree was constructed using 157 NBS domains. Percent bootstrap values (1000 iterations) are indicated in every branch. Each protein is encoded as follows: NB + ID number (same as phytozome ID) + Domains present (TNL, TN, N_TIR_, NL_TIR_, CNL, CN, N_CC_, NL_CC_) + chromosome assignment (if available). Red, green, purple, and blue correspond to TIR, CNL-2, RPW8, and CNL-1 clades, respectively. APAF-1 was used as an outgroup.

**Figure 2:**
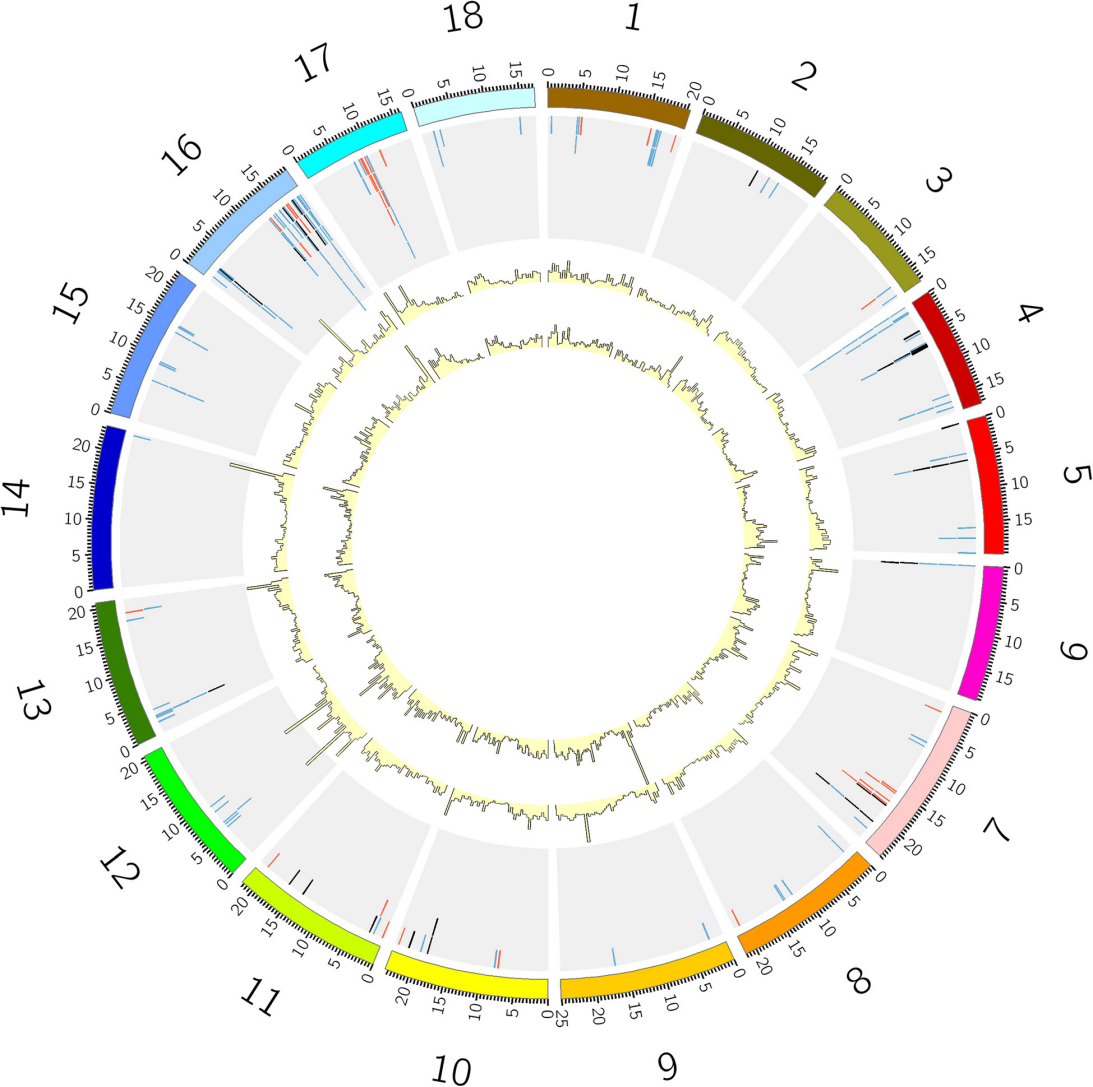
Distribution of Cassava predicted NBS-LRR resistance genes. The relative map position of 205 NBS-LRR genes is shown on each of the 18 cassava chromosomes. Each gene is represented by a colored tick mark. TNL genes are represented by red ticks, while CNL and partial genes are represented by blue and black tick marks, respectively. NBS-LRR clusters are evident on chromosomes 16 and 17. In the center, gene expression densities in healthy cassava leaves (outer histogram) and roots (inner histogram) depict transcriptional activity of the NBS-LRR rich regions. Expression densities were obtained by mapping RNASeq reads to the genome and were plotted using 0.5 Mb windows.

To study the evolutionary relationships among the newly discovered NBS-LRR genes, we built a phylogenetic tree using the conserved NB-ARC domain. Predicted NBS-LRR genes that contained no or partial NB-ARC domain were excluded. Alignment of the amino acid sequences revealed the NBS-subdomains, including the p-loop, kinase-2, kinase-3, and GLPLA. This alignment also showed a previously reported diagnostic site [58] that can differentiate CNL and TNL proteins right after the kinase-2 sub-domain (Additional file 5). As expected, the phylogenetic tree separated TNL and CNL genes into two different clades (Figure 1). For clarity, we labelled the genes with their type and chromosome position. The TNL clade is comprised of 33 genes, including several incomplete genes (NL_TIR_, N_TIR_). These genes are distributed among nine chromosomes (Figures 1 and 2), with a relatively high density on chromosome 17. On the other hand, the CNL clade has three main groups: CC(I), CC(II), and a separate clade that includes those proteins encoding an RPW8 (Resistant to powdery mildew in *A. thaliana*) domain. This strong separation has not been observed in previous studies where the RPW8s genes grouped together with the CC(II) group [29].

**Figure 3:**
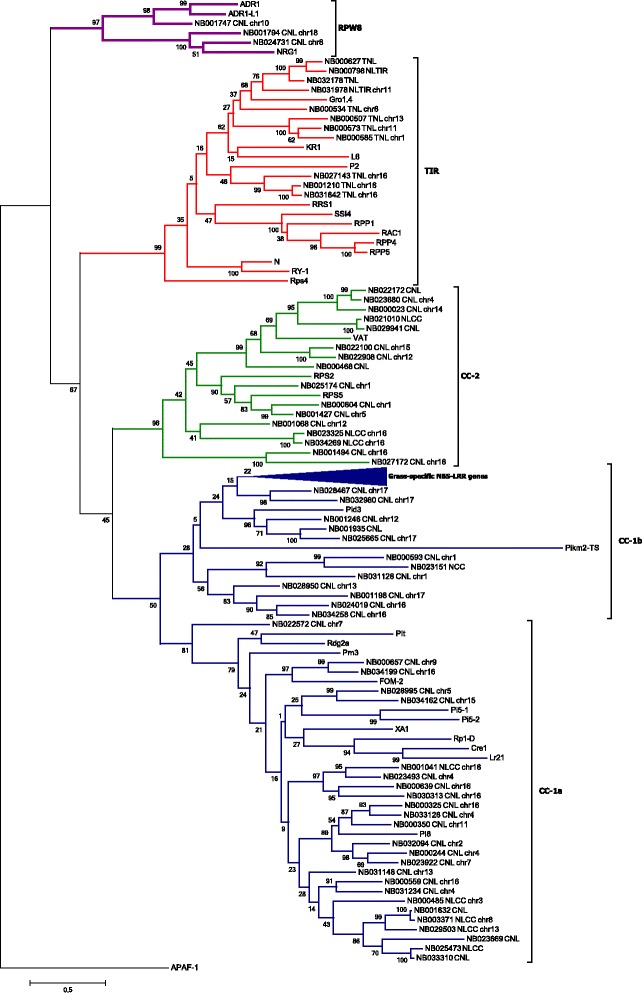
Phylogeny of a subset of cassava NBS-LRR proteins with functional resistance genes from other species. Phylogenetic analysis of the NBS domain was carried out by the Maximum likelihood method using cassava NBS-LRR proteins as well as cloned functional R genes from different species including *Arabidopsis*, rice, and more (Additional file 3). For ease of visualization, we only used a subset of cassava genes that represents each clade (see Additional file 6 for the full tree). Red, green, purple, and blue represent TIR, RPW8, CNL-2, and CNL1 clades, respectively. The bottom clade in the CNL1-1b group is compressed, because it groups resistance proteins that are specific to grasses and does not cluster together with any cassava NBS-LRR protein. Members of this clade include *Pi* genes from *Oryza sativa*, *Lr* genes from *Triticum aestivum,* and *Mla* genes from *Hordeum vulgare,* for example.

For comparative purposes, we included well characterized and manually curated resistance genes from *Arabidopsis thaliana*, *Cucumis melo*, *Hordeum vulgare*, *Solanum tuberosum,* and *Zea mays,* among others (Additional file 3) into a second phylogenetic tree (Figure 3, Additional file 6). Most of the clades grouped as previously observed. All the TNL reference genes grouped into the TNL cluster (red), including Gro1.4 (*Solanum tuberosum*), N (*Nicotiana glutinosa*), and KR1 (*Glycine max*); however, these proteins tended to cluster separately from other TNL cassava genes. RPS4 (*Arabidopsis thaliana*), for example, clustered separately from all the other TNL members, as was previously reported in potato [30].

The CC-1 clade (blue) harbored more than half of the total NBS-LRR genes, and most of the reference R genes clustered inside this group as well. The introduction of the reference genes, however, influenced the topology of the tree, and resulted in a division within this group into two separate clades, CC-1a and CC-1b (Figure 3, Additional file 6). CC-1a grouped 58 cassava CNL genes. As was observed inside the TNL group, most of the reference R genes tended to cluster apart from the cassava resistance candidates, although two functionally validated genes, FOM-2, (*Cucumis melo*) and Pl8 (*Helianthus annuus*), showed sequence similarity to some cassava genes. FOM-2 clustered together with NB000657 and NB034199 with high bootstrap support, while PI8 was part of a sub-branch that contained several cassava genes. The CC-1b clade had 27 cassava genes; when adding the reference R genes, the topology of this subgroup broke apart (Additional file 6). Most of the reference genes in this clade belonged to grass species (*Hordeum vulgare*, *Oriza sativa,* or *Triticum aestivum*) and, thus, it was not a surprise that none of these clustered together with any cassava genes. The NBS-LRR family in grasses has a markedly different evolution that is represented by a significant underrepresentation of TNL genes [59].

The RPW8 clade (purple), containing three cassava genes, clustered with two ADR1 genes from *Arabidopsis* and the N-required gene 1 (NRG1) from *Nicotiana benthamiana*. Two sub-clades were evident; one contained ADR1 genes and NB001747, and the other contained NRG1, NB001794, and NB024731.

The last clade, CC-2 (green, previously reported as CNL-B, [22]), contained 36 cassava members. Only three reference genes fell into this group: Virus aphid Transmission (VAT, from *Cucumis melo*) and Resistance to *Pseudomonas syringae* protein 5 and 2 (RPS5 & RPS2). This group was previously reported as part of the CCR clade [29], although we did not obtain the same result.

### Gene mapping

Physical chromosomal positions were established for 205 (~63%) of the NBS-LRR genes (the rest are on unanchored scaffolds) using their nucleotide sequences and the v5 assembly, and visualized using Circos and Map Chart (Figure 2, Additional file 7, Additional file 8). CNL genes were present on all the cassava chromosomes with at least one representative, while the distribution of TNL genes was more limited, having genes on only 9 chromosomes (Figure 2). We must consider, however, that 37% of the genes remain unmapped, so these estimates may be inaccurate.

**Figure 4:**
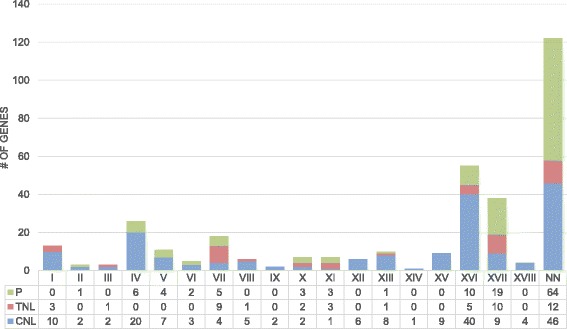
TNL, CNL, and partial genes distribution. Gene distribution for each class is shown across the cassava chromosomes. Bars are divided into CNL genes (blue), TNL genes (red), and partial genes (green).

It is clear (Figure 2, Figure 4) that the distribution of NBS-LRR genes is not even among the chromosomes and that they tend to form clusters. This clustered arrangement has been thought to facilitate sequence exchange through recombinational mispairing [34]. To identify NBS-LRR clusters, we used a previous definition [29] that a NBS-LRR cluster has two or more genes that are closer than 200 kb and separated by no more than eight non-NBS-LRR genes. Using this approach, we identified 39 clusters containing 143 NBS-LRR genes. Thus, 62 (30%) are singleton genes that do not map near other resistance genes. The size of the clusters varied across the genome from 2 to 10 members; the clusters can be classified further as homogeneous or heterogeneous based on how related the members of each cluster are (Additional file 9).

Chromosome 16 has the highest number of R genes (40, ~20% of mapped genes) distributed in 9 clusters plus 9 singletons. The number of members per cluster in this chromosome varies from 2 to 10. Cluster 35, for example, contains 10 genes belonging to the CC-1a clade (Figure 1, Additional file 9) with homology to RGA-2 (Resistance protein to *P. infestans* in tomato and potato). Multiple sequence alignment, followed by phylogenetic tree reconstruction of the proteins that belong to that group, shows that there are two subgroups within the cluster that represent two different origins (Additional file 9). Cluster 31 also carries 10 NBS-LRR genes, and belongs to the CC-2 clade with homology to putative resistance genes. There are only five TNL proteins in this chromosome, and only two of them are close enough to be considered a cluster by our criterion (cassava4.1_031642m and cassava4.1_001210m); these proteins encode close homologs to the TMV resistant protein N.

We only observed TNL clusters in chromosomes 7, 16, and 17. Most of the clusters comprise paralogs derived from the same recent common ancestor. It is less common to find TNL proteins clustered together with CNL proteins. In chromosome 17, for example, we found two neighbouring clusters (37 and 38) that encoded TNL genes; the first cluster carried 6 members of the TNL group with homology to TMV resistant protein N, and the second cluster contained 3 TNL and 6 CNL proteins. Two of the TNL genes lacked some domain (N_TIR_, TN) and were very short, 633 and 480 bp, respectively, and the remaining gene, cassava4.1_027701m (975 bp), appeared to be a pseudogene caused by a frameshift mutation. While these TNL proteins might be the remnants of previously functional genes that were defeated by pathogens, we cannot exclude the possibility of a sequencing/annotation error. The CNL proteins in the cluster belong to the CC-1b clade and are closely related.

We also checked the genome distribution of the RPW8-NBS-LRR proteins (purple clade in Figure 2). Three proteins belong to this clade, NB001747 (homologous to ADR1 *Arabidopsis* gene), NB001794, and NB024731 (homologous to NRG1 from *Nicotiana*). Genes that encode these proteins are distributed on chromosomes 10, 18, and 8, respectively, with sizes that range from 794 to 828. None of these genes are located close to another resistance protein. All the members of this clade have strong homologs in *Populus trichocarpa*, *Riccinus communis,* and *Jatropha curcas*.

### Expression of NBS-LRR genes under biotic stresses

Recently a study on changes in the cassava transcriptome under Cassava Brown Streak Virus (CBSV) infection [53] found no significant differential expression of NBS-LRR genes in the Cassava genome one year after infection, either in a resistant or susceptible genotype. Only 235 NBS-LRR genes were identified in this study (based on conserved domains), contrasting with our finding of 327. We also found that some of the genes called as NBS-LRR in that study were miss-annotated and belonged to members of other families. FPKM (Fragments Per Kilobase of exon per Million fragments mapped) values were obtained for the 327 NBS-LRR genes that we found and, confirming the observation of Maruthi et al., we saw no significant changes in expression between the infected and the control plant (Additional file 10).

Similar results were obtained in the plants infected with *Xanthomonas axonopodis* pv manihotis (causal agent of Cassava Bacterial Blight). In this experiment only one partial NBS-LRR cassava gene (cassava4.1_006209m) was differentially expressed during infection with the pathogen. There are however high expression of several of this genes across all conditions suggesting that they may still have a role in cassava’s response to these pathogens.

## Discussion

Cassava is a staple crop for millions of people in Africa, being their primary source of calories (FAO, 2003). This crop has a high yield potential under good conditions [60], yet it faces many biotic stresses. Given the importance of cassava, breeding for disease resistance is essential; the availability of the recently published cassava genome sequence allowed us to identify, classify, and map the NBS-LRR members, the biggest disease resistance gene family in plants.

According to our bioinformatics analysis the cassava genome carries a total of 327 NBS-LRR genes. From these, we annotated 99 as partial NBS-LRR genes that encode none or only a small part of the NBS domain, but they have high similarity with full-sized NBS-LRR genes. Partial genes may be the result of pseudogenization, given the rapid evolution of this gene family, but we cannot eliminate the possibility that these genes were incorrectly annotated because we did not perform a manual re-annotation to examine for sequencing errors. The 327 NBS genes found in the cassava reference genome represent 0.9% of the total number of coding sequences. The frequency of NBS sequences in the cassava genome fall within the range previously observed for other species (0.6% - 1.76%) [61].

No functional resistance genes have been cloned in cassava; genes found in this study, however, have strong homology with previously reported cassava Resistance Gene Candidates (RGCs) and NBS-LRR genes from other species. [35,58]. Lopez et al. [58] reported 12 Resistance Gene Candidates (RGC) in the cassava genome. The sequences for nine of these RGC regions were made available publicly. Eight of the nine RGCs aligned with >90% identity to NBS-LRR genes found in this study (Additional file 11). Additionally, the same study reported an RGC cluster at the end of linkage group J using a BAC library and RGC6 sequence as a probe. This region corresponded to the top of chromosome 4, which we found carries an NBS-gene cluster that contains the closest RGC6 homolog: cassava4.1_023508m (Additional file 12). More recently, Gedil et al. [35] reported the sequence of several Resistance Gene Analogs (RGA) from different cassava varieties. All of the sequences reported as NBS-LRR-like were associated with 13 NBS-LRR sequences found in this study (Additional file 11).

Association studies and QTL identification for disease resistance are scarce in cassava. Most of these are related to Cassava Bacterial Blight (CBB), which is caused by different strains of the pathogen *Xanthomonas axonopodis* (*Xam*) [62-65]. One of these QTLs, which is associated with resistance to *Xam* strain CIO151 exclusively, explained 61% of the phenotypic variance and was located in linkage group U [65]. While the molecular marker associated with this QTL was not available, a nearby CAPS marker, DR11, is located at position ~16 Mb of chromosome 16, in the center of the largest NBS-LRR supercluster found in this study. Another major effect QTL was reported that confers resistance to Cassava Mosaic Disease (CMD) on cassava chromosome 8 [66]. This region, however, lacks any mapped NBS-LRR gene. More association studies on different diseases using different cassava genotypes may reveal a role for genes and clusters that we detected in this study.

Of the 228 full-length NBS-LRR genes, 181 belong to the CNL class, and 47 to the TNL class. This means that there are 3.8× more CNL than TNL genes. This ratio is indeed variable, and Leister (2004) [67] suggested that the over-representation of one of these groups could reflect the adaptation of the R genes to the predominant pathogens. For example, in *Oriza sativa* and *Sorghum bicolor*, members of the TNL family are present in a low frequency of approximately 1% [24,68]. In general, most grasses analysed contain only a few or no TNLs [59,69,70], which suggests that this class is specific for dicotyledons [28]. It is also interesting that most CNL genes from grasses presented in this study have no homologs among dicots (Figure 3, Additional file 6), which demonstrates that the evolution of NBS-LRR genes diverged significantly between monocots and dicots. Species of Brassicaceae, however, have a high percentage of TNLs: *Arabidopsis thaliana* (64%) and *Brassica rapa* (64%) [18,22]. Finally, there are some examples of ratios similar to what we found: in grapevine, for example, the proportion of CNL over TNL proteins is 3.8× [26] and in potato that ratio increases to 4.7× [29]. The over-representation of CNL in potato may be because CNL genes are typically responsible for resistance to *Pythopthora infestans* [29]. It was expected that the evolution of this family would be tightly linked with the pathogens affecting each species. Moreover, the rapid evolution of these genes may be visible among different cultivars from the same species in environments with different biotic stresses.

Previous studies showed that the CNL group forms two phylogenetic clades, the canonical one and the CNL-R group, including members that encode an RPW8 domain in their N-terminal region. It is interesting, however, that the CNL branch in cassava does not include the RPW8 clade. We found that RPW8 genes were strongly separated from all other CNL genes, which was supported by strong bootstrap results (Figure 1, Figure 3). The RPW8 clade was described previously and referred to as CNL-A [22] or the CCR-NB-LRR encoding genes [71]. This family is thought to be one of the most ancestral of the major CC-NB-LRR clades [22], and it has been suggested to work differently than the more common CNL genes [71-73]. The ADR1 gene, present in this clade, is known to be an atypical CNL gene from *Arabidopsis,* which encodes abnormally conserved LRR domains and two conserved additional motifs in the NBS surroundings [74]. The homology and conservation of motifs is evident among proteins of this group, as shown by MEME (Additional file 13).

We tried to find close homologs from a set of known functional resistance genes (Figure 3, Additional file 6) for members of every clade, but there are a significant number of branches, especially in clade CC-2, that show no significant similarity to any of these well characterized genes. These genes might provide resistance to unknown cassava pathogens or may play a role in non-host resistance responses [75].

As mentioned previously, the cluster arrangement of NBS-LRR genes is considered to facilitate rapid gene evolution [76]. Several mechanisms have been proposed to contribute to the genomic diversity and distribution of this gene family: intragenic and unequal crossovers, gene conversion, positive and diversifying selection, and tandem duplications [67]. Most of the cassava NBS-LRR genes (70% of mapped genes) are located within a cluster; the biggest cluster is located in chromosome 16 with 10 CNL members. In homogeneous clusters like this, expansion is associated with tandem duplications. While most clusters are comprised of closely related genes, there are exceptions where members belong to different phylogenetic lineages; cluster 38 is an example, within which we found members of both TNL and CNL families. The formation of these heterogeneous clusters is thought to be the result of transposition, ectopic recombination, or chromosomal translocations [77]. As suggested before, this kind of genome evolution may be the result of positive selection for a higher complexity that can serve as the basis of new NBS-LRR – pathogen effector specificities [28,78].

In an effort to clarify the “cluster” definition, simulations were conducted to determine if the distribution observed in the NBS-LRR genes was caused by chance (see Methods). We observed that clusters of 2 and 3 genes occurred at the same frequency in a random sample of genes than when analysing NBS-LRR genes (Additional file 14). For clusters containing more than 4 members, the difference in frequencies is clear, which suggests that, at least for cassava, only clusters with 4 or more members might be significant.

While the definitions used to detect clusters might be arbitrary, cassava NBS-LRR genes tend to lie in more evident superclusters, such as the 43 NBS genes on the end of chromosome 16 and the 19 genes in the middle of chromosome 17 (Figure 2). Collectively, these genes represent more than 30% of the total number of mapped NBS-LRR genes. Superclusters have been observed in other plants such as *Arabidopsis*, rice, and *Medicago*. In *Medicago*, an NBS-LRR supercluster represents more than 5% of all the genes present in the upper arm of the chromosome where it is located. In this scenario, the authors suggested that NBS superclusters may have played an important role in genomic remodelling during the evolution of those chromosome regions [16].

It is interesting that a high percentage of NBS-LRR genes are expressed constitutively in cassava leaves (72%). Moreover, 77% of the partial genes that might be considered as pseudogenes exhibit evidence of an RNAseq expression. Whether these genes have an actual function or whether their expression is a temporary genome drag remains unclear. While not analysed in this study, the percentage of pseudogenes in NBS-LRR genes in plants can be very high. In rice, it was found to be as high as 55% [79] Truncated NBS-LRR genes are often located close to intact NBS-LRR genes and are also clustered on specific chromosomes [16,30], a pattern that is followed commonly by the partial genes in cassava (black on Figure 2). The function of NBS-LRR pseudogenes are not well defined; they are usually only considered as genes that will be eliminated from the genome or sources of genetic diversity that may be used through recombination [19]. However, there may be a larger role for these genes. For example, in mice an expressed pseudogene played a role in maintaining the stability of its full-length homolog mRNA by interfering with the local silencing system [80]. In plants, truncated NBS-LRR peptides produced by alternative splicing (similar to what expressed pseudogenes look like) have a role in promoting disease resistance [81]. Uncovering the function of these expressed pseudogenes would be a major step to fully understanding plant-pathogen interactions.

Recently, studies of the cassava transcriptome under CBSV [53] and CBB [55] infection reported no significant differential expression of NBS-LRR genes among different time points and cassava genotypes. The lack of upregulation of these genes during infection is not surprising, and there are several explanations for this behaviour. Resistance to CBSD and CBB is considered to be quantitative and multigenic [1,53], so that NBS-LRR genes may not be involved in the resistance phenomena at all. While this is a possibility, we should also consider that many NBS-LRR genes are expressed constitutively, meaning that NBS gene products will be present already in the plant cells to promote resistance even before the infection. Under this second scenario, NBS-LRR genes are not necessarily over-expressed to act in disease resistance. Additionally, when comparing susceptible and tolerant genotypes, gene expression might not be as relevant as the presence/absence of the specific resistance allele. We have to consider that the reference cassava genotype, AM560-2, is a partially inbred line derived from the Latin-American cassava cultivar MCOL-1505, that may lack NBS-LRR genes present in other genotypes. Moreover CMD and CBSV are recent diseases specific to Africa and are not present in the center of cassava domestication; comparing this analysis with some African genotypes would be valuable to see if evolution has caused divergence in the NBS-LRR gene family. Finally, high throughput methodologies, such as Resistance gene enrichment sequencing (RenSeq) [82] coupled with QTL or GWAS studies for other cassava diseases, would allow us to start mapping NBS-LRR clusters to specific pathogens.

## Conclusions

We have identified 228 NBS-LRR type genes plus 99 partial genes related to the same family in the cassava genome. Information on the phylogeny of these genes and, most importantly, their physical positions on the chromosomes represent a valuable tool in future efforts to identify novel functional resistance genes in different cassava genotypes and other *Manihot* species. High throughput genotyping can also serve to explore the diversity of these regions across different genotypes. This kind of analysis would help decipher the recent evolution and dynamics of NBS-LRR genes in this clonally propagated crop.

## Additional files

Additional file 1:
**NBS domain identification pipeline.** The process used for the identification of proteins encoding and NBS domain using hmm-pfam is presented. Additional file 2:
**NBS-associated conserved domains identification pipeline.** The process used for the identification of NBS-associated conserved domains using hmm-pfam is presented. Additional file 3:
**List of reference R genes from different species.** This file contains the list of the functional R genes proteins used as reference in the phylogenetic trees. Plant Resistance Gene database (prgdb.crg.eu/wiki) IDs, names, donor species, and protein type are shown. Additional file 4:
**List of identified NBS-LRR genes.** Includes the ID for all the NBS-LRR genes annotated in this study with additional information. Size of the protein in amino acids, best hit against the *Arabidopsis* genes, top hit after blast against the UNIREF 100 database, family code, domains present in the gene, chromosome assignment, and the code used for the phylogeny analysis. Additional file 5:
**NBS multiple alignment and subdomain conservation.** A subset of CNL and TNL NBS domains was aligned to show the conserved subdomains; p-loop, kinase-2, kinase-3, and GLPLA. Additional file 6:
**Phylogenetic tree plus reference R genes.** A tree was calculated using the same parameters as in Figure 2, but using all the cassava NBS-LRR genes that carry a full NBS domain. Additional file 7:
**Position of anchored NBS-LRR genes and cluster assignment.** This file shows the genome position and cluster assignment of all the anchored genes across the 18 cassava chromosomes. Additional file 8:
**Detailed position of each NBS-LRR gene on the chromosomes.** TNL genes are shown in red, CNL on blue, and partial genes on black. Additional file 9:
**CNL cluster with 10 members.** The 10 genes are clustered together in a ~500 kb region. While the genes are very similar overall, there are two different sources of evolution (Red and Blue) as shown by DNA alignments b) and average distance tree. c) It is counterintuitive that members of red and blue groups are physically mixed. Moreover, the different “strand orientation” of the genes represents the complexity of evolution within NBS-LRR genes. Additional file 10:
**FPKM values for NBS-LRR genes during CBSV infection.** Expression values of NBS-LRR genes for control and CBSV infected plants are shown for two different cassava genotypes. Additional file 11:
**Homology between NBS-LRR genes previously reported.** Two different sets of NBS-LRR genes previously reported for casssava (Lopez et al. [58]; Gedil et al. [35]) were compared to the ones found in the present study using Blast. Additional file 12:
**NBS-LRR cluster co-localizing with previously reported cluster.** Eleven NBS-LRR homologs found in the tip of chromosome 4 share the same position as the previously proposed NBS-LRR cluster in linkage group J (Lopez et al. [58]). Additional file 13:
**RPW8 motif conservation as revealed by MEME.** Conserved motifs are shown as inferred by MEME. RPW8 genes show a remarkable conservation of the motifs that encode LRR domains (motifs 5, 6, 8, and 4). Additional file 14:
**Stochastic cluster assignment simulation.** Number of clusters per size were calculated for both the NBS-LRR genes and a simulated data set consisting of 1000 iterations of 205 random cassava genes.

## Abbreviations

NBS-LRR: Nucleotide Binding-site and leucine-rich repeat
CC: Coiled-coil
TIR: Toll/interleukin 1 receptor
APAF-1: Human apoptotic protease-activating factor-1
CNL: CC-NBS-LRR
TNL: TIR-NBS-LRR
FPKM: Fragments per kilobase of exon per million fragments mapped
CBSD: Cassava Brown Streak Disease
CBSV: Cassava Brown Streak Virus
CBB: Cassava Bacterial Blight
HMM: Hidden Markov Model
QTL: Quantitative Trait Loci
