# Supplementary figures and images for: Identification and distribution of the NBS-LRR gene family in the Cassava genome
Source: BMC Genomics. 2015 May 7;16(1):360. doi: 10.1186/s12864-015-1554-9 (PMC4422547; doi:10.1186/s12864-015-1554-9)

# Additional file 1.- Identification of NBS-LRR pipeline

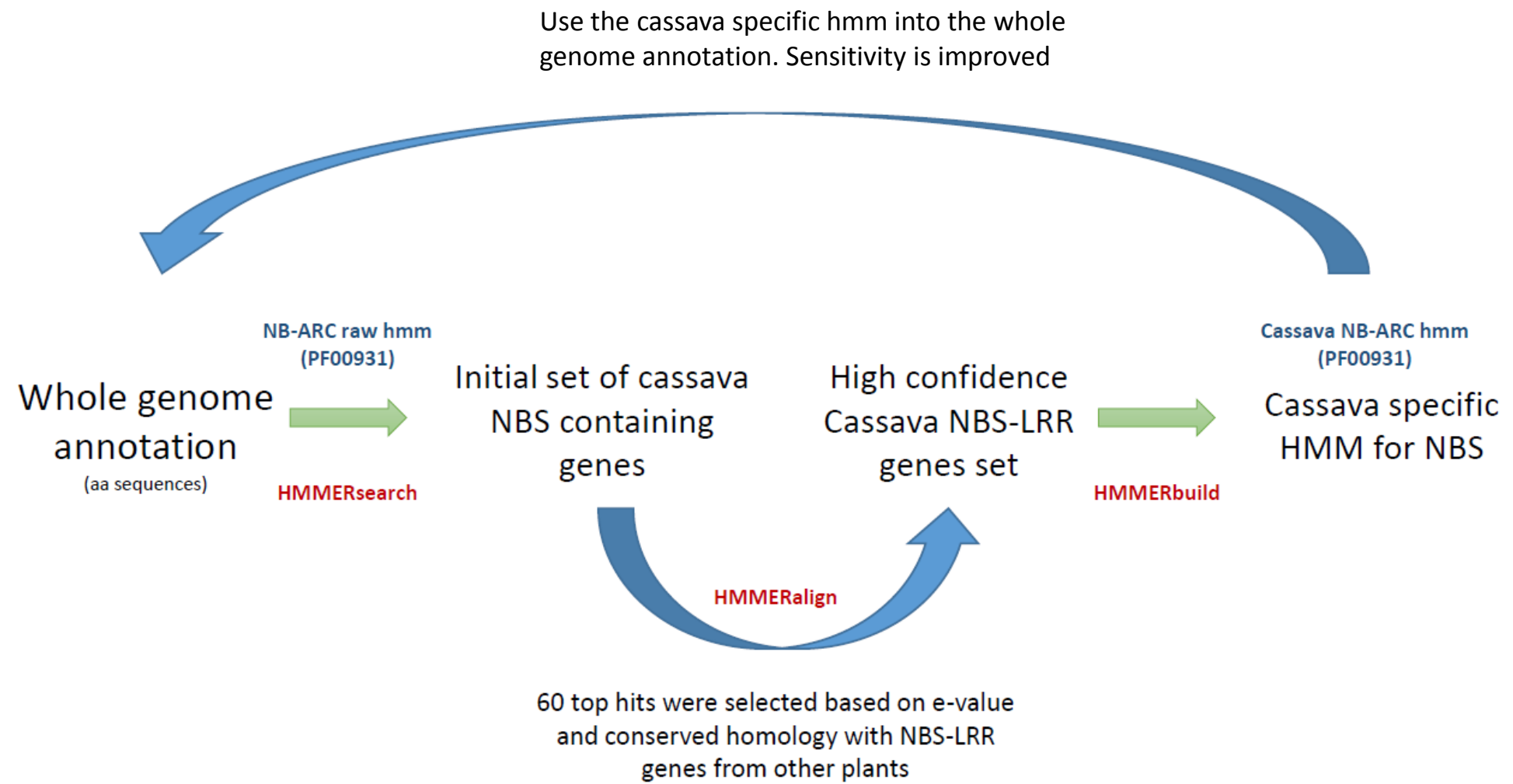

Supplement: Additional file 1: — NBS domain identification pipeline. The process used for the identification of proteins encoding and NBS domain using hmm-pfam is presented. [file 12864_2015_1554_MOESM1_ESM.pdf]

**Additional file 2.-** Identification of NBS-LRR-associated conserved domains pipeline

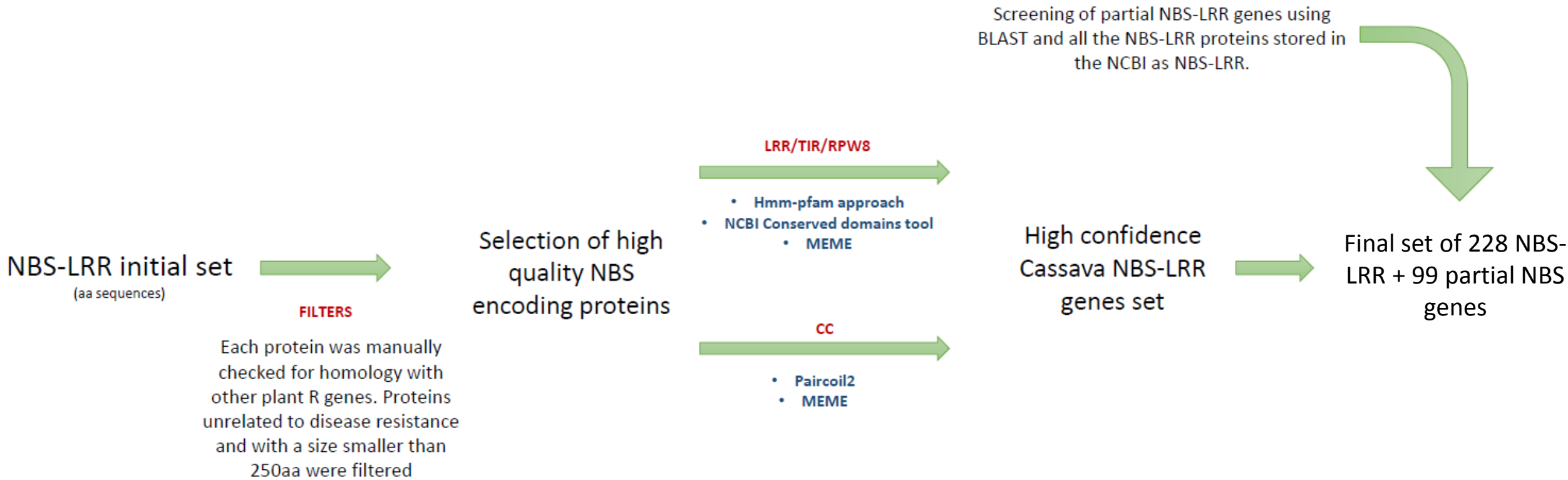

Supplement: Additional file 2: — NBS-associated conserved domains identification pipeline. The process used for the identification of NBS-associated conserved domains using hmm-pfam is presented. [file 12864_2015_1554_MOESM2_ESM.pdf]

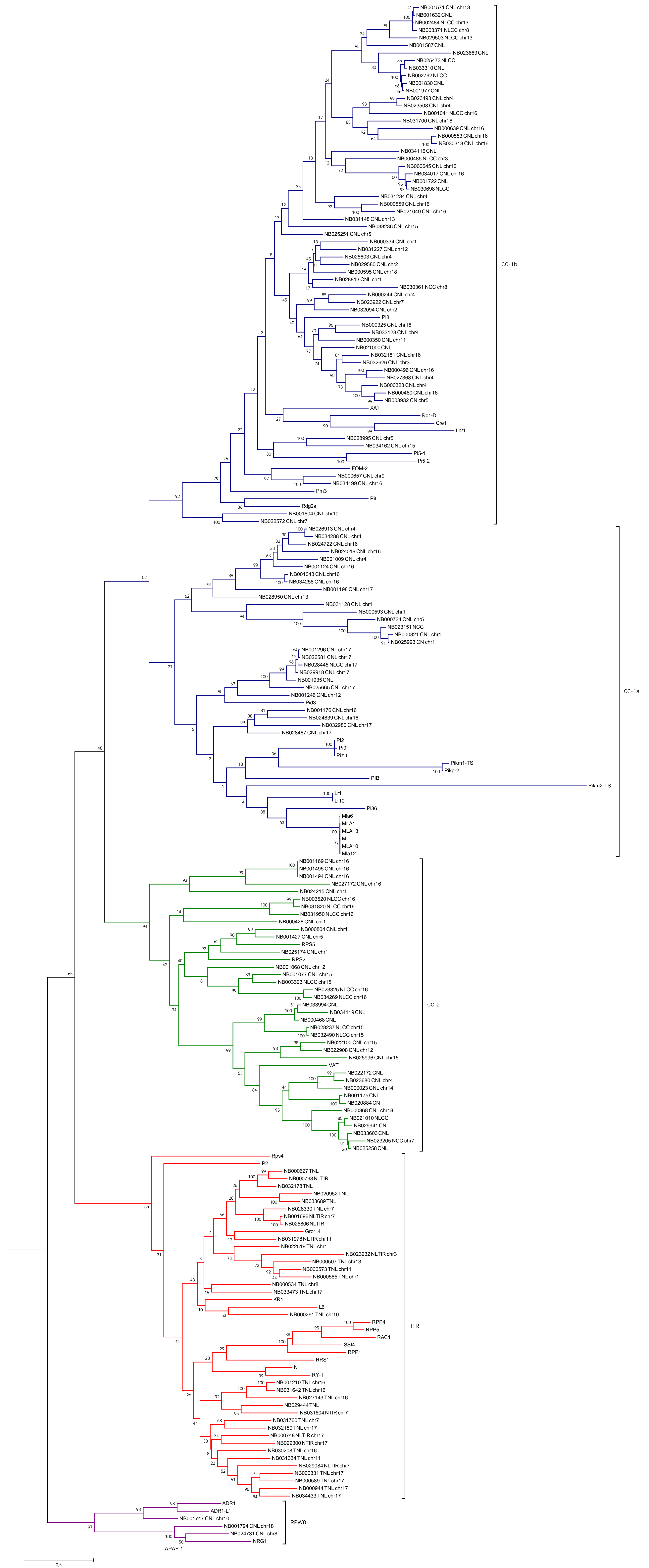

Supplement: Additional file 6: — Phylogenetic tree plus reference R genes. A tree was calculated using the same parameters as in Figure 2, but using all the cassava NBS-LRR genes that carry a full NBS domain. [file 12864_2015_1554_MOESM6_ESM.pdf]

## Slide 1
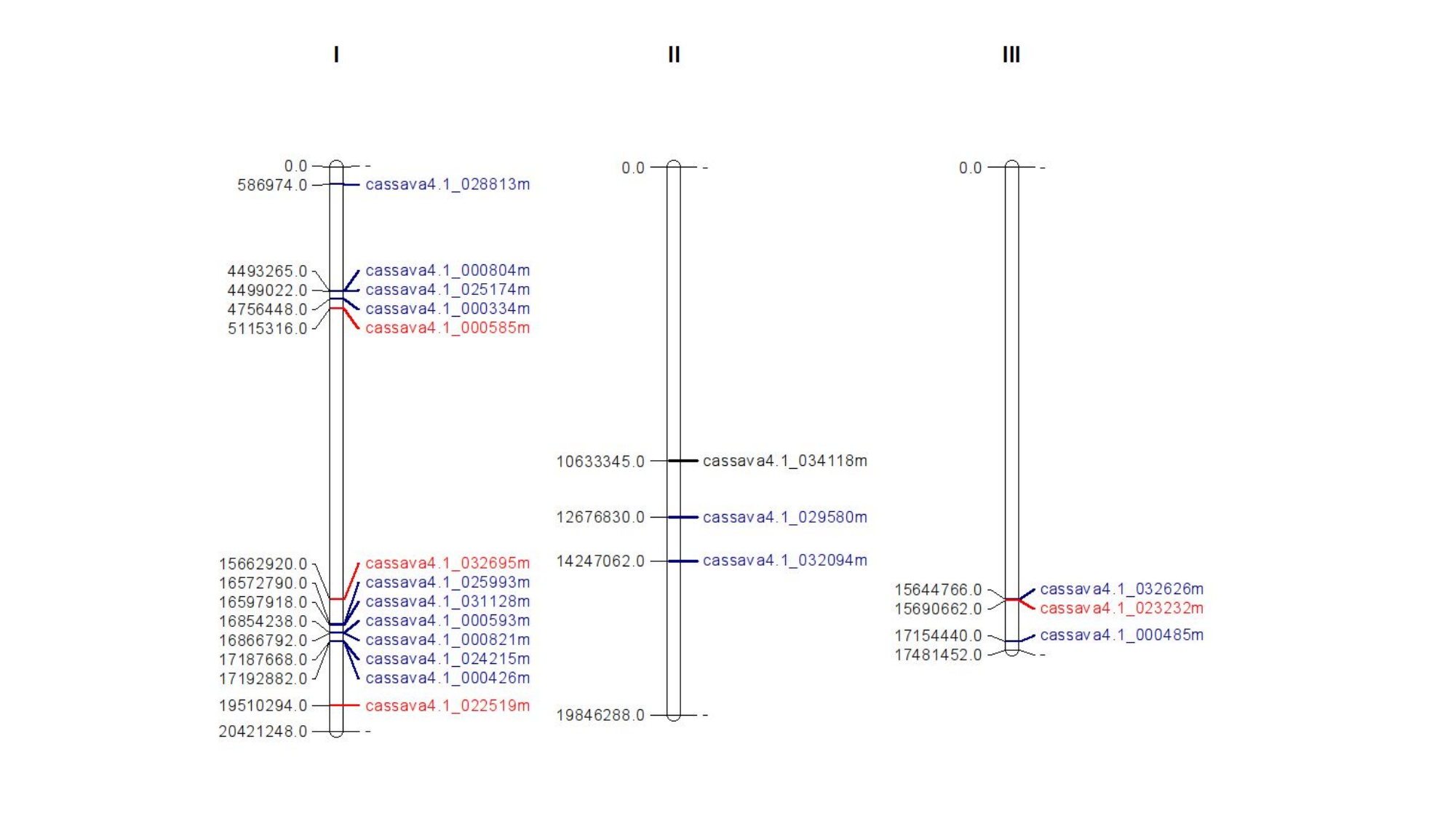

## Slide 2
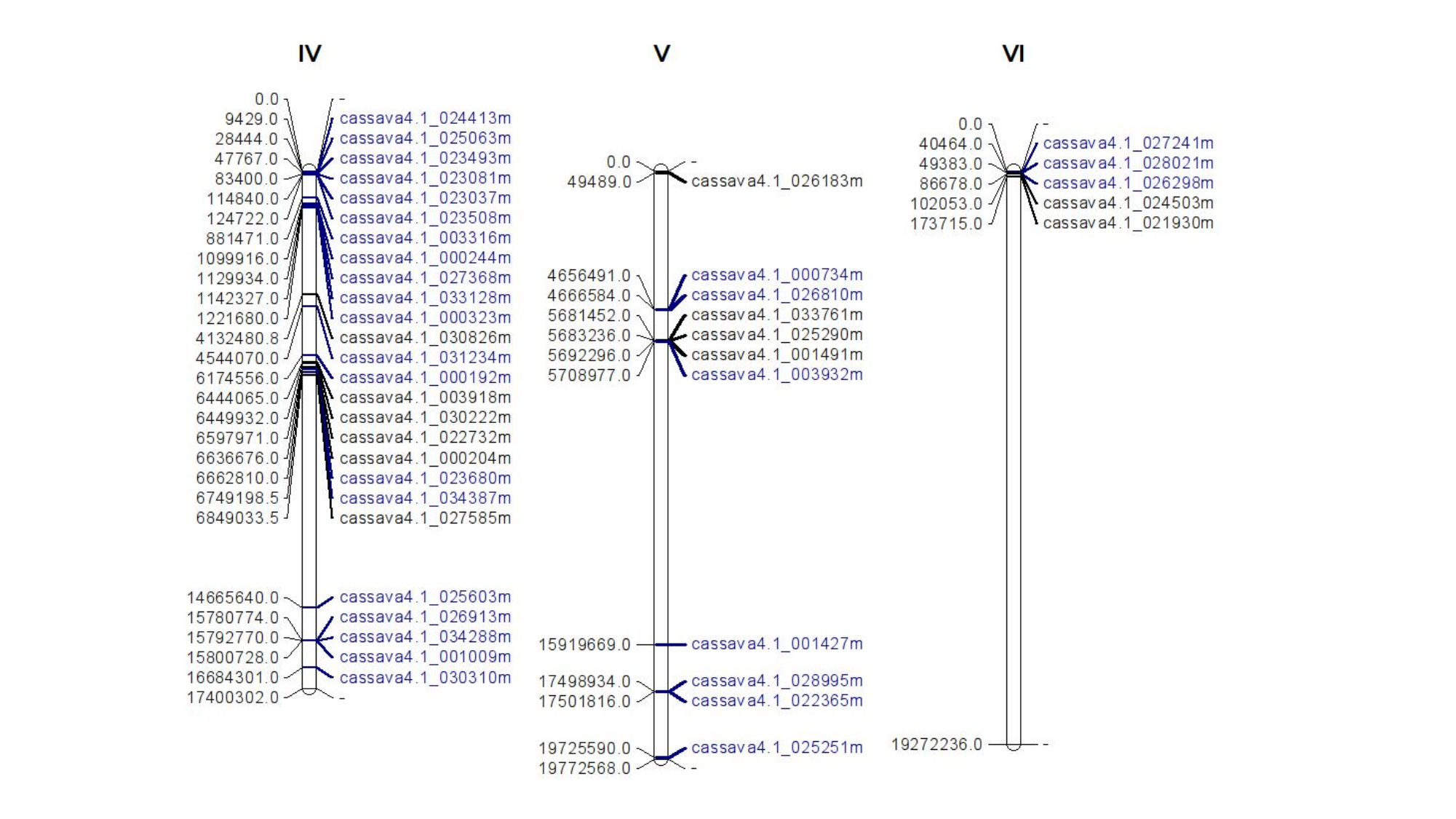

## Slide 3
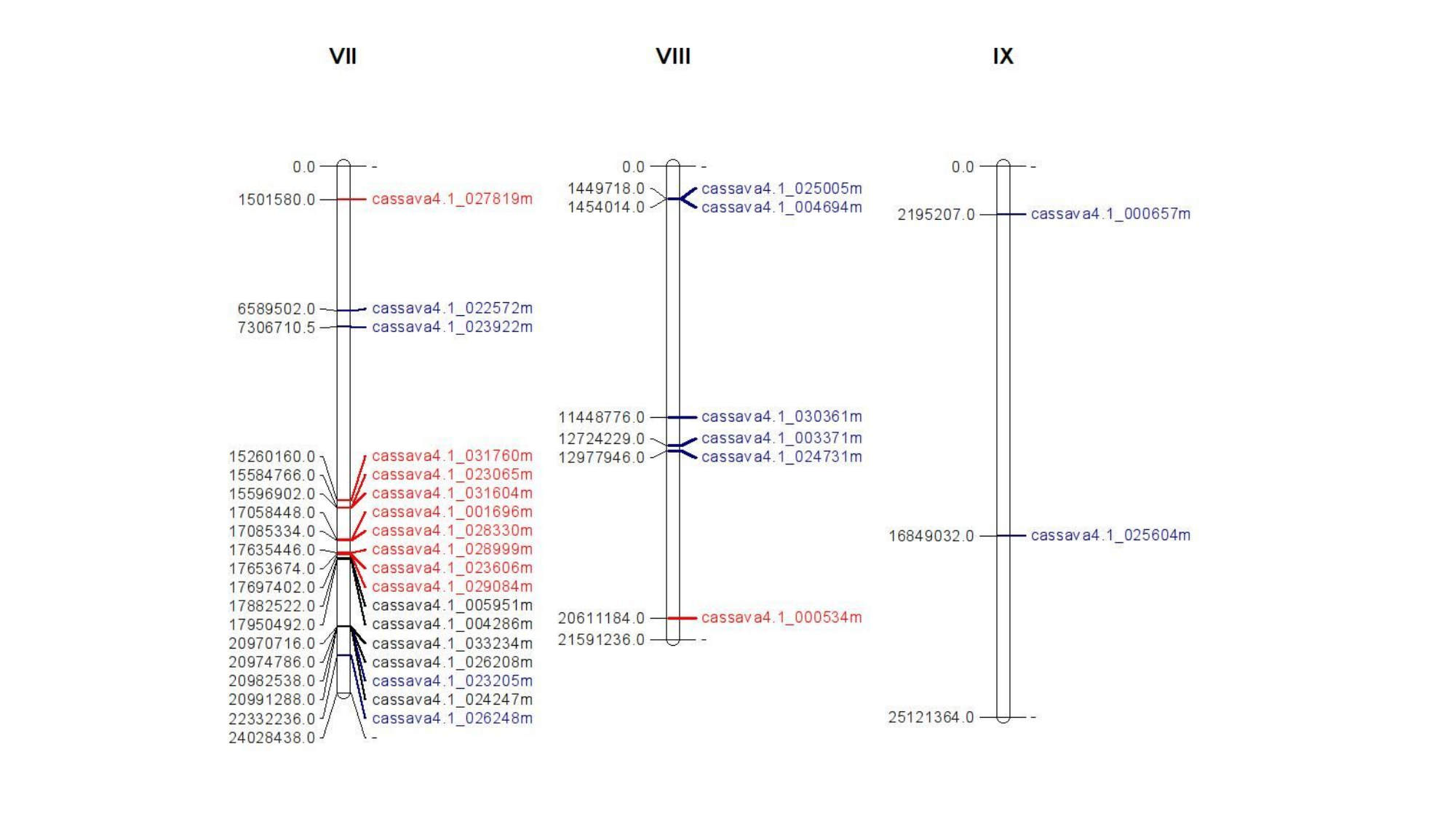

## Slide 4
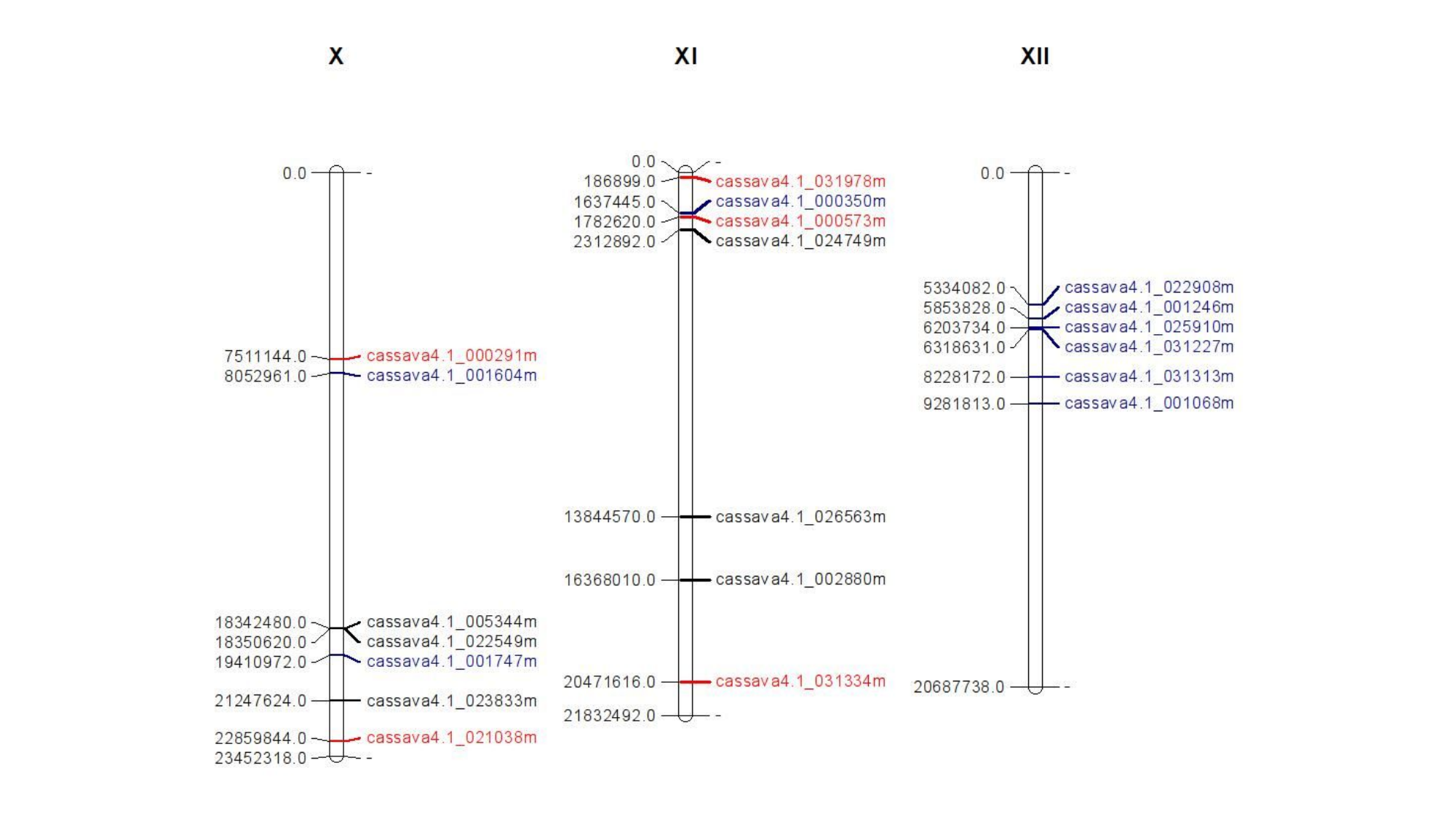

## Slide 5
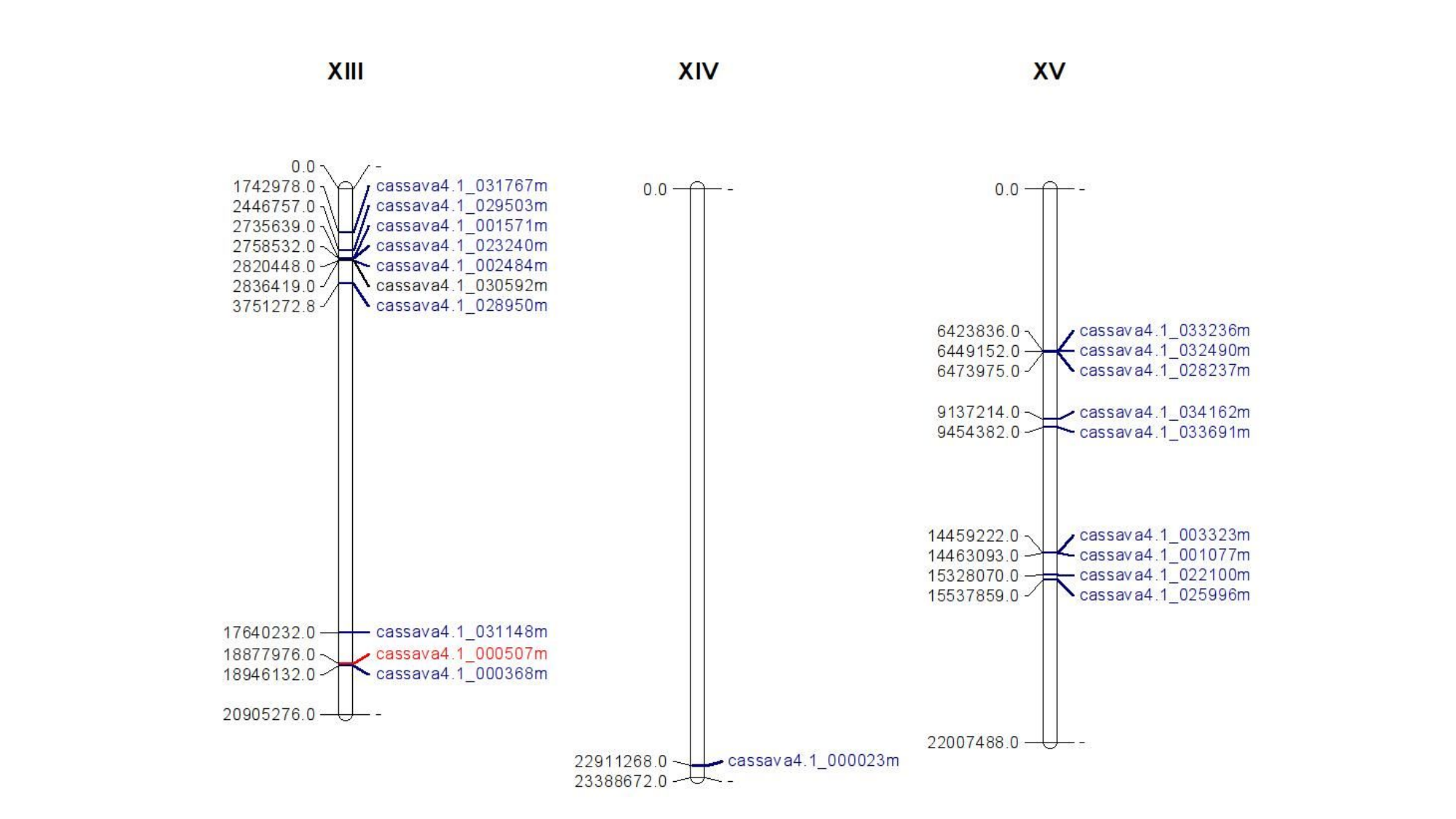

## Slide 6
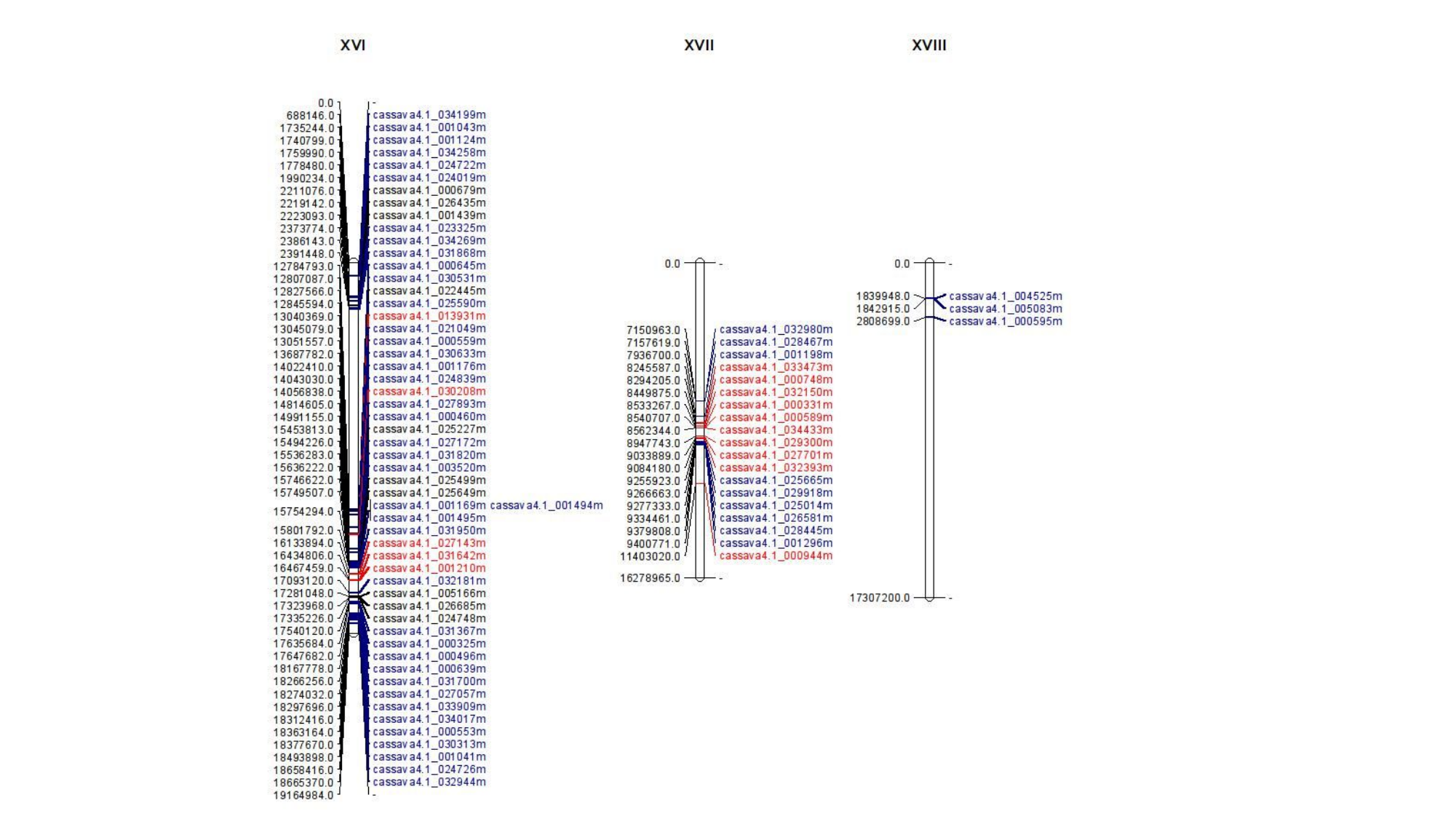

Supplement: Additional file 8: — Detailed position of each NBS-LRR gene on the chromosomes. TNL genes are shown in red, CNL on blue, and partial genes on black. [file 12864_2015_1554_MOESM8_ESM.pptx]

## Slide 1
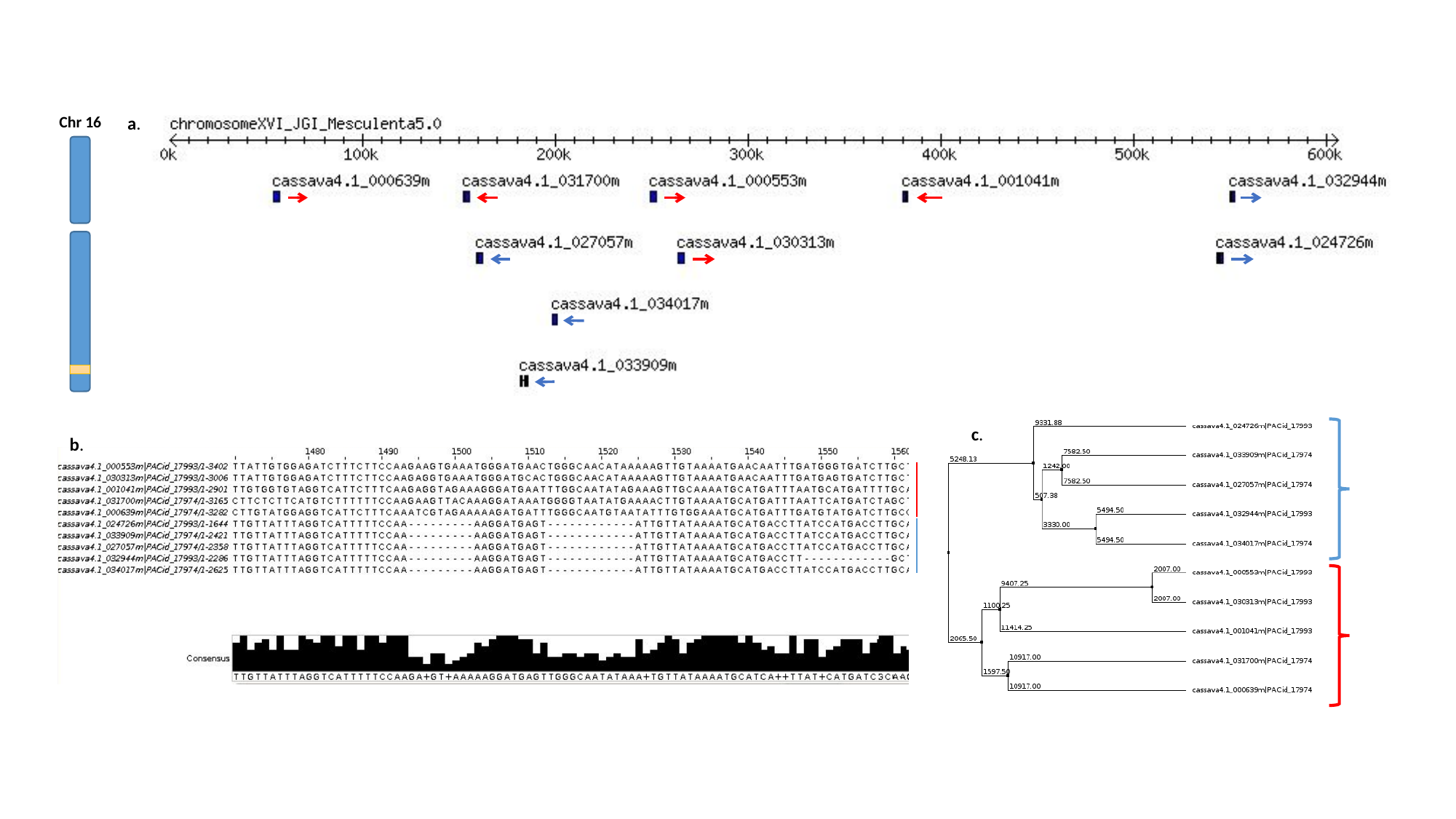

Chr 16
a.
c.
b.

Supplement: Additional file 9: — CNL cluster with 10 members. The 10 genes are clustered together in a ~500 kb region. While the genes are very similar overall, there are two different sources of evolution (Red and Blue) as shown by DNA alignments b) and average distance tree. c) It is counterintuitive that members of red and blue groups are physically mixed. Moreover, the different “strand orientation” of the genes represents the complexity of evolution within NBS-LRR genes. [file 12864_2015_1554_MOESM9_ESM.pptx]

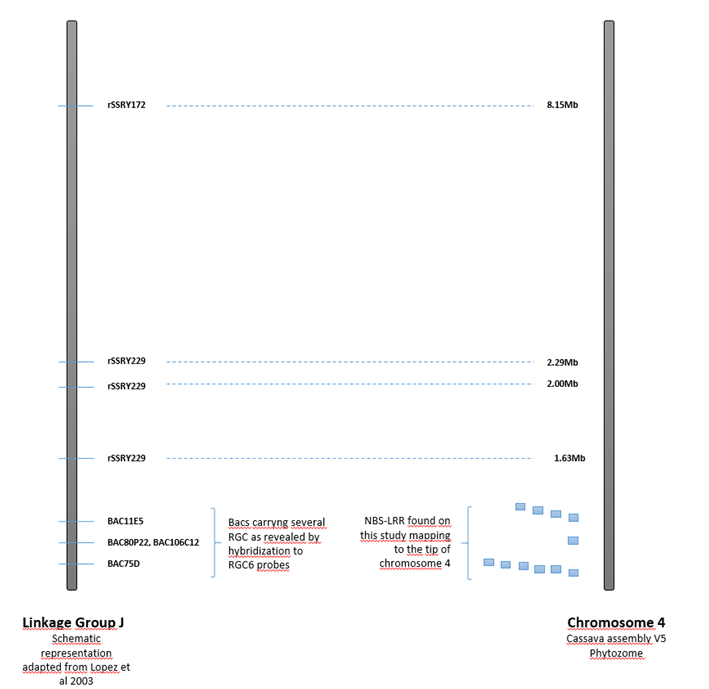

Supplement: Additional file 12: — NBS-LRR cluster co-localizing with previously reported cluster. Eleven NBS-LRR homologs found in the tip of chromosome 4 share the same position as the previously proposed NBS-LRR cluster in linkage group J (Lopez et al. [58]). [file 12864_2015_1554_MOESM12_ESM.png]
