# Supplementary material for: Identification and distribution of the NBS-LRR gene family in the Cassava genome
Source: BMC Genomics. 2015 May 7;16(1):360. doi: 10.1186/s12864-015-1554-9 (PMC4422547; doi:10.1186/s12864-015-1554-9)
Supplement: Additional file 5: — NBS multiple alignment and subdomain conservation. A subset of CNL and TNL NBS domains was aligned to show the conserved subdomains; p-loop, kinase-2, kinase-3, and GLPLA. [file 12864_2015_1554_MOESM5_ESM.pptx]

## Slide 1
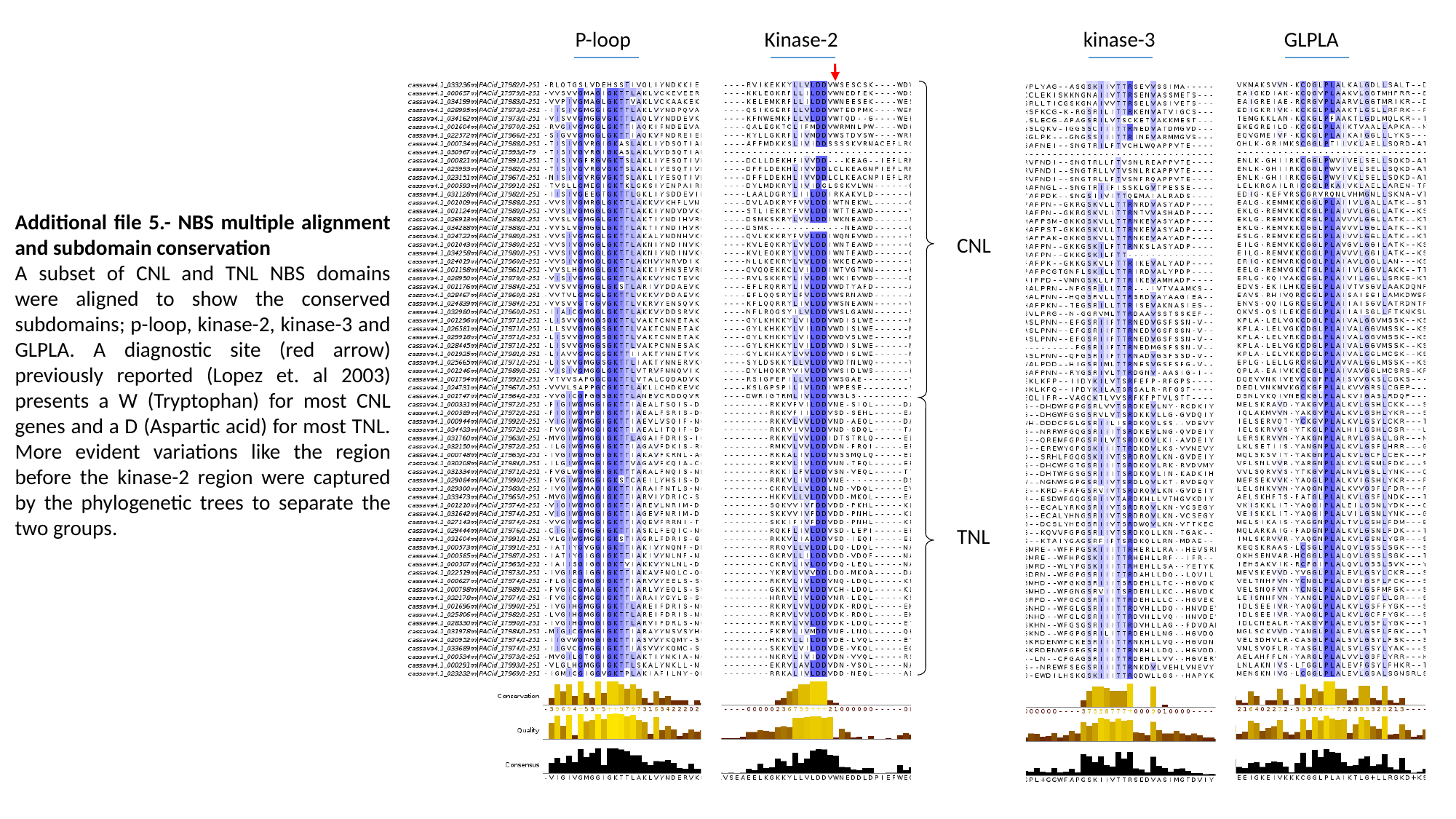

kinase-3
GLPLA
Kinase-2
P-loop
Additional file 5.- NBS multiple alignment and subdomain conservation
A subset of CNL and TNL NBS domains were aligned to show the conserved subdomains; p-loop, kinase-2, kinase-3 and GLPLA. A diagnostic site (red arrow) previously reported (Lopez et. al 2003) presents a W (Tryptophan) for most CNL genes and a D (Aspartic acid) for most TNL. More evident variations like the region before the kinase-2 region were captured by the phylogenetic trees to separate the two groups.
CNL
TNL
