# Supplementary material for: Identification and distribution of the NBS-LRR gene family in the Cassava genome
Source: BMC Genomics. 2015 May 7;16(1):360. doi: 10.1186/s12864-015-1554-9 (PMC4422547; doi:10.1186/s12864-015-1554-9)
Supplement: Additional file 13: — RPW8 motif conservation as revealed by MEME. Conserved motifs are shown as inferred by MEME. RPW8 genes show a remarkable conservation of the motifs that encode LRR domains (motifs 5, 6, 8, and 4). [file 12864_2015_1554_MOESM13_ESM.pptx]

## Slide 1
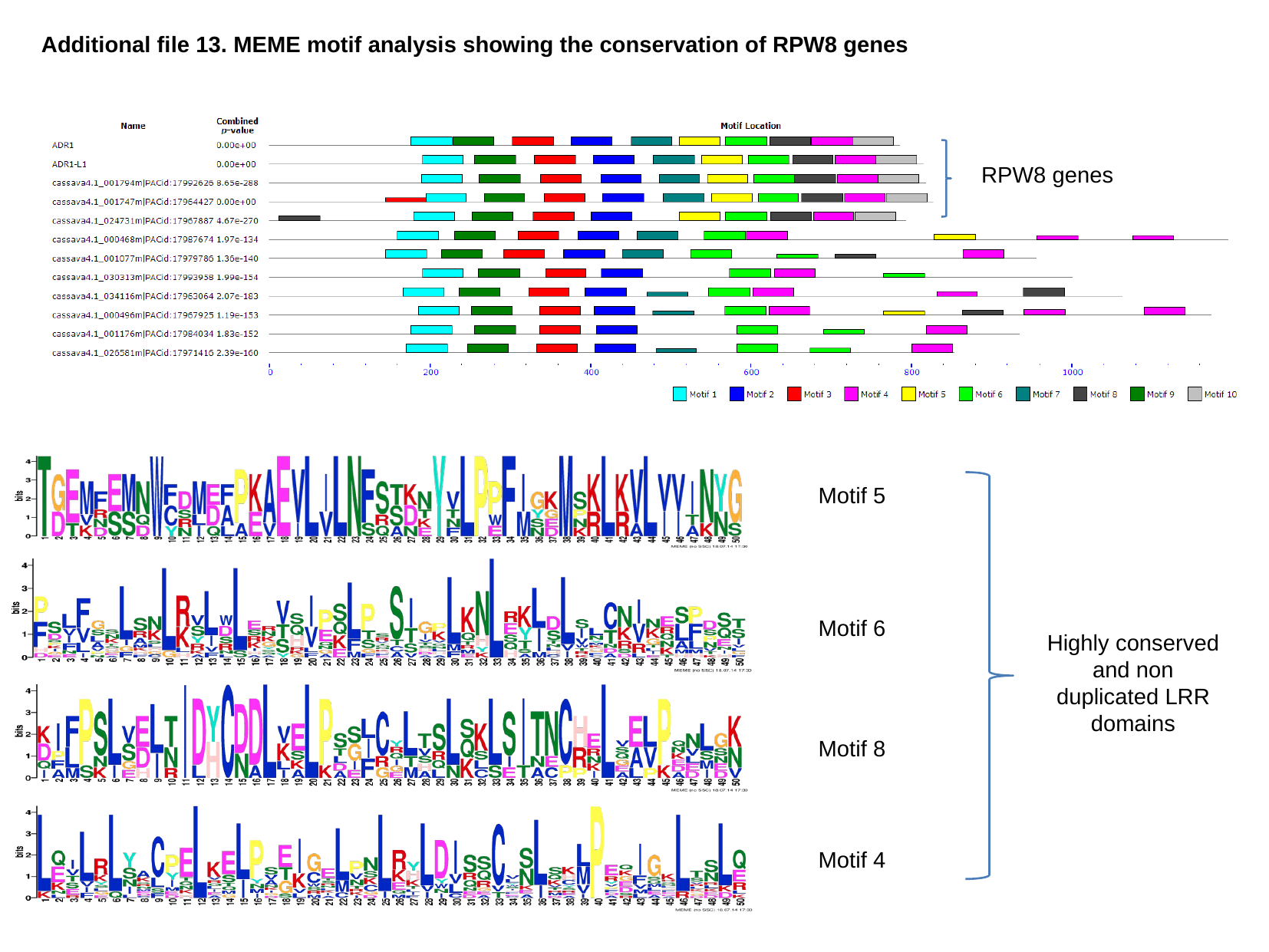

Additional file 13. MEME motif analysis showing the conservation of RPW8 genes
RPW8 genes
Motif 5
Motif 6
Highly conserved and non duplicated LRR domains
Motif 8
Motif 4
